# The Expression of Placental 17β-Hydroxysteroid Dehydrogenase Genes Is Associated with the Elevation of Active Androgens and Estrogens in Pregnant Women, but Does Not Affect 11-Oxygenated C19 Steroids

**DOI:** 10.3390/ijms27104290

**Published:** 2026-05-12

**Authors:** Yuko Yokohama, Yugo Watanabe, Ke-ichi Nakajima, Akihiro Umezawa, Satoru Takahashi, Yasuhiro Mori, Yasuhito Kato, Jun-ichi Kawabe, Takashi Yazawa

**Affiliations:** 1 Department of Gynecology and Obstetrics, Asahikawa Medical University, Asahikawa 078-8510, Hokkaido, Japan; 2 Department of Biochemistry, Asahikawa Medical University, Asahikawa 078-8510, Hokkaido, Japan; 3 National Center for Child Health and Development Research Institute, Setagaya 157-8535, Tokyo, Japan; 4 Department of Pediatrics, Asahikawa Medical University, Asahikawa 078-8510, Hokkaido, Japan; 5 Mori Hospital, Asahikawa 070-0037, Hokkaido, Japan

**Keywords:** placenta, androgen, estrogen, 11-oxygenated androgen, HSD17B1

## Abstract

The placenta produces a variety of steroid hormones through the catalytic activity of steroidogenic enzymes, including cytochrome P450 (CYP) hydroxylases and hydroxysteroid dehydrogenases (HSD). Large amounts of progesterone produced by the placenta are essential for the maintenance of pregnancy. Although androgens and estrogens are also elevated in maternal circulation during gestation, there are conflicting reports on whether de novo synthesis of these steroids occurs in the human placenta. To address this issue, we performed a comprehensive analysis of steroidogenic gene expression in early and term placenta. While none of the genes examined showed binary expression changes, 17β-HSDs, including HSD17B1 and AKR1C3, were markedly upregulated in the term placenta. CYP19A1 and HSD11B2 genes were also markedly upregulated. In contrast, CYP17A1, CYP21A2, CYP11B1, CYP11B2, and HSD17B3 were almost undetectable. Consistent with these findings, the plasma ratios of active to precursor sex steroids (estradiol/estrone and testosterone/androstenedione) were higher in pregnant than in non-pregnant women, although concentrations of all steroids increased. In contrast, plasma levels and profiles of 11-oxygenated androgens were unchanged. These results indicate that the human placenta does not significantly contribute to circulating levels of either classical or novel classes of androgens. Therefore, this study provides new insights into the tissue of origin and the physiological significance of sex steroids during gestation.

## 1. Introduction

**Figure 1:**
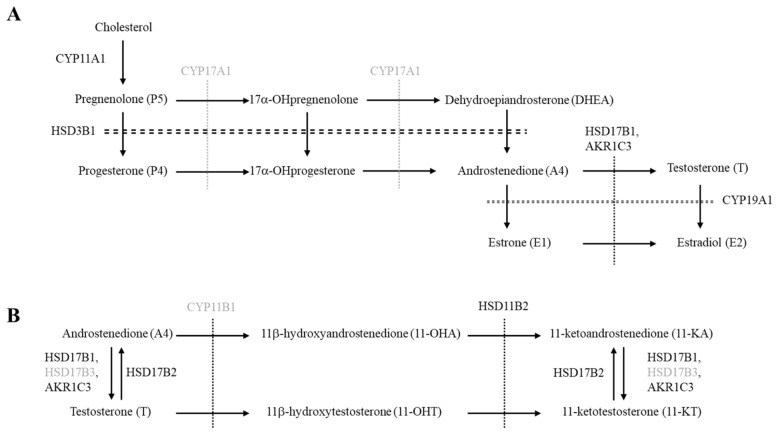
Pathways for steroid hormone production in the human placenta. (**A**) Pathway for producing classical sex steroids. (**B**) Pathway for producing 11-oxygenated androgens from androstenedione (A4). Enzymes not expressed and pathways not active in the human placenta are indicated by gray font and dashed lines, respectively.

The placenta produces a variety of steroid hormones that play important roles in the maintenance of pregnancy [1]. Steroid hormones in the placenta are synthesized from cholesterol through a series of reactions catalyzed by steroid cytochrome P450 (CYP) hydroxylases and hydroxysteroid dehydrogenases (HSD). This is also the case in the primary steroidogenic organs, such as the testis, ovary, and adrenal (Figure 1) [2,3]. However, the intracellular cholesterol transport system in the human placenta is markedly different from that of the primary steroidogenic organs. In the gonads and adrenal glands, steroidogenic acute regulatory protein (StAR) is responsible for the rate-limiting step in steroidogenesis by regulating cholesterol transport from the outer to the inner mitochondrial membrane. StAR expression is known to be pituitary hormone-dependent [4]. In contrast, intracellular cholesterol transport in the placenta is constitutively active through the STARD3 protein [4,5,6]. This difference is likely due to the fact that the placenta must continuously produce large amounts of progesterone to inhibit uterine contraction and prevent preterm labor. Placental progesterone is produced from cholesterol in a series of steps by cytochrome P450 family 11 subfamily A member 1 (CYP11A1) and 3β-HSD type I (HSD3B1) [6].

The placenta also produces estrone (E1) and estradiol (E2) in a species-specific fashion [7]. In humans, these estrogens are not synthesized de novo in the placenta due to CYP17A1 deficiency. Instead, they are derived from dehydroepiandrosterone (DHEA) and its metabolites, which are predominantly produced by the fetal adrenal. DHEA is converted into weak sex steroids, androstenedione (A4) and E1, through the activity of HSD3B1 and CYP19A1. These weak sex steroids are then converted into the strong sex steroids, testosterone and E2, by HSD17B1 [7], although testosterone itself can also serve as a precursor of E2. It has been proposed that placental E2 production protects the pregnant woman and fetus from virilization caused by androgen excess [8,9]. Although placental CYP19A1 deficiency causes virilization of the mother and female fetus in utero due to impaired conversion of androgens to estrogens, mothers deliver the infants at term in almost all cases.

However, in contrast to the traditional concept, several recent studies have reported that the human placenta expresses CYP17A1 and autonomously produces androgens [10,11,12]. These studies hypothesized that placenta-derived androgens contribute not only to the elevation of maternal plasma estrogens as their precursors, but also to the elevation of plasma A4 and testosterone. In addition to these classical androgens, 11-oxygenated C19 steroids (11-oxygenated androgens), such as 11β-hydroxyandrostenedione (11-OHA), 11-ketoandrostenedione (11-KA), 11β-hydroxytestosterone (11-OHT), and 11-ketotestosterone (11-KT), represent a novel class of androgens in human [13,14]. They are mainly produced in the adrenal from A4 and testosterone through the activities of CYP11B1, HSD11B2, and HSD17B5 (aldo-keto reductase 1C3, AKR1C3) enzymes [15]. Because the placenta lacks the expression of CYP11B1, de novo synthesis of 11-oxygenated androgens does not occur [16]. However, the human placenta contains much higher levels of 11-KA and 11-KT than A4 and testosterone, respectively [17]. These findings suggest that the placenta may contribute to fluctuations in maternal 11-oxygenated androgen levels by metabolizing adrenal-derived 11-OHA and 11-OHT, in a manner similar to how it metabolizes DHEA into classical androgens and estrogens. To address these possibilities, we investigated placental expression of steroidogenesis-related genes and plasma steroid hormone concentrations in pregnant women.

## 2. Results

### 2.1. Expression of Placental Steroidogenic Genes During Gestation

**Figure 2:**
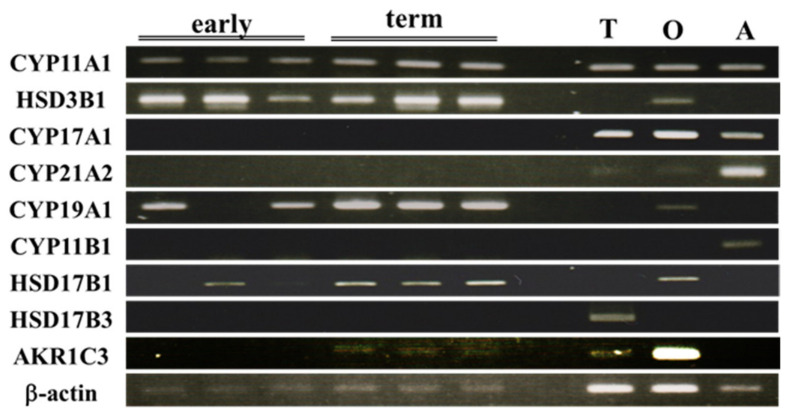
Expression of steroidogenic genes in human placenta during gestation. RT-PCR analyses of each gene in the placenta at the first trimester (early) and at full term (term). Testis (T), ovary (O), and adrenal (A) were used as positive controls.

**Figure 3:**
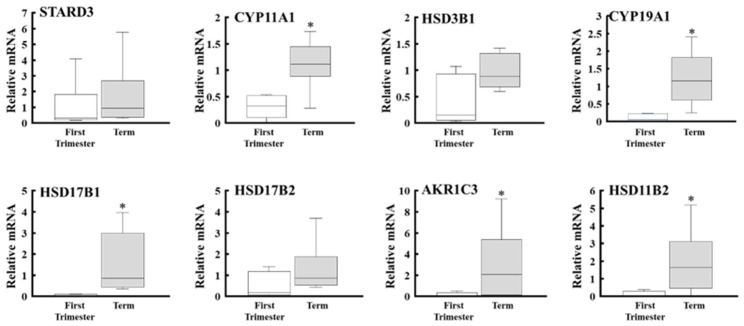
Comparison of expression levels of steroidogenic genes between the first trimester and full term placenta. mRNA expression of each gene in each placenta tissue sample (*n* = 8) was analyzed by qPCR and normalized to β-actin. Bars within boxes indicate the median. Boxes represent the interquartile range (25th–75th percentiles), and whiskers indicate the full range of the data. *p*-values were calculated using the Wilcoxon rank-sum test to assess differences between groups. * *p* < 0.05 vs. early (first trimester).

We compared the expression of placental steroidogenic genes between early pregnancy and full term by RT-PCR and qPCR (Figure 2 and Figure 3). None of these genes showed on/off expression changes, although the expression levels of some genes fluctuated during gestation. CYP11A1, HSD3B1, CYP19A1, HSD17B1, AKR1C3, and HSD11B2 genes were expressed at both stages. qPCR analyses showed that the expression of CYP11A1, CYP19A1, HSD17B1, AKR1C3, and HSD11B2 genes was significantly increased at full term compared with early pregnancy (Figure 3). In addition to other steroidogenic genes (HSD3B1 and HSD17B2), the expression level of the cholesterol deliverer STARD3 gene was similar at both stages. In contrast, CYP17A1, CYP21A2, CYP11B1, CYP11B2, and HSD17B3 genes were almost undetectable at both stages.

### 2.2. Fluctuation of Plasma Sex Steroid Profiles During Pregnancy

**Figure 4:**
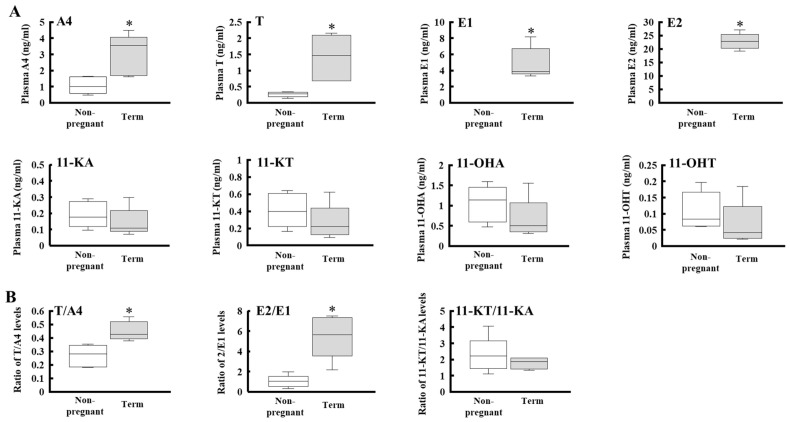
Comparison of plasma androgens and estrogen levels in non-pregnant women and pregnant mothers. (**A**) Plasma A4, testosterone (T), E1, E2, 11-KA, 11-KT, 11-OHA, and 11OHT levels were measured by LC-MS/MS. (**B**) Comparison of plasma ratio of active sex steroids (T, E2, and 11-KT) to weak steroid precursors (A4, E1, and 11-KA) between non-pregnant women and pregnant mothers. Bars within boxes indicate the median. Boxes represent the interquartile range (25th–75th percentiles), and whiskers indicate the full range of the data. *p*-values were calculated using the Wilcoxon rank-sum test to assess differences between groups. * *p* < 0.05 vs. non-pregnant women.

Among the upregulated genes at full term, the elevation of 17β-HSD genes (HSD17B1 and AKR1C3/HSD17B5) was remarkable. Because these enzymes are involved in the production of active sex steroids, we compared the profiles of plasma sex steroids in non-pregnant women and preterm mothers (Figure 4 and Table 1). Consistent with previous studies, both androgens and estrogens were elevated in pregnant women compared with non-pregnant women, and the elevation in estrogen levels was much greater than that of androgen levels (A4, 2.82-fold; testosterone, 5.39-fold; E1, 154.9-fold; E2, 545.2-fold). Although the levels of both precursors and active steroids were increased, the elevation of latter steroid levels was remarkable. In fact, the ratios of testosterone/A4 and E2/E1 in pregnant women were significantly increased compared with those in non-pregnant women. Testosterone/A4 ratio was 0.28-fold in non-pregnant women and 0.43-fold in pregnant women. E1 and E2 levels were almost the same in non-pregnant women, whereas E2 was higher than E1 by 5.7-fold in pregnant women. In contrast to the classical androgens and estrogens, 11-oxygenated androgens were not affected by gestation. The levels of all four 11-oxygenated androgens were not significantly changed (Figure 4 and Table 1). Consistent with these findings, the 11-KT/11-KA ratio was almost constant.

## 3. Discussion

The human placenta produces a variety of steroid hormones. Among them, progesterone production is maintained at high levels throughout gestation, whereas E2 production is markedly increased with the progression of pregnancy [18]. Consistent with these findings, our study shows that the expression of progesterone-producing enzymes (CYP11A1 and HSD3B1) with cholesterol deliverer STARD3 was almost constant, whereas expression of E2-producing genes (CYP19A1 and HSD17B1) was markedly increased at late pregnancy. In contrast, CYP17A1 was undetectable. Although some studies have indicated CYP17A1 expression and de novo androgen synthesis in the human placenta [10,11,12], our results support the widely accepted concept that the human placenta lacks CYP17A1 and therefore cannot synthesize androgens de novo. These findings suggest that de novo progesterone synthesis is the dominant steroidogenic function of the human placenta during gestation. This is reasonable as pregnancy maintenance in humans depends on progesterone production, which shifts from luteal to placental sources during early gestation. A comparable luteo–placental shift occurs in horses, where the placenta shows little or no CYP17A1 expression [19]. In addition, this result supports the traditional view that human placental estrogen synthesis relies predominantly on fetal adrenal-derived DHEA-S, with only a minor contribution from the maternal adrenal [2,3,6,20]. Therefore, it is conceivable that the elevation of E2 in maternal plasma is driven not only by increased fetal adrenal DHEA production associated with adrenal maturation [21], but also by the induction of placental CYP19A1 and HSD17B1 expression. In particular, high HSD17B1 expression levels increase the E2/E1 ratio, even though both estrogens are markedly elevated in pregnant women. However, the present study only assesses steroidogenic gene expression at the transcript level. Protein expression and enzymatic activity were not evaluated, and further studies are necessary to determine whether these transcriptional changes translate into functional differences.

In contrast to the classical sex steroids, plasma levels of 11-oxygenated androgens did not increase during gestation. Some studies reported that concentrations of 11-oxygenated androgens are higher than those of classical androgens in full-term placenta [17]. Among them, 11-KA was the most abundant in both male and female placentas. This phenomenon is likely due to high HSD11B2 expression. However, as in non-pregnant women, 11-OHA is the most abundant 11-oxygenated androgen in pregnant women [22]. In addition, 11-KT levels are higher than those of 11-KA. These results suggest that 11-oxygenated androgens in the maternal blood are mainly derived from adrenal, as in non-pregnant women [23,24]. It is also conceivable that androgen production by maternal adrenal does not increase during gestation, although cortisol production is increased by elevation of ACTH levels [25]. This phenomenon might be due to placental estrogens inhibiting adrenal androgen production. In pregnant baboons, estrogen administration suppresses serum DHEA and DHEAS levels in both intact and fetectomized mothers, whereas serum cortisol levels were unaffected [26]. Reduction in bioactive androgens derived from the adrenal is also reported in patients taking estrogen replacement therapy [27]. However, the molecular mechanisms are still unclear. Our study has some limitations, particularly the small sample size (*n* = 5). In fact, contrary to our results, He and colleagues reported that plasma 11-KA levels are elevated in pregnant women and neonatal cord blood [28]. In contrast, Karahoda and colleagues recently found no such increase in maternal and neonatal serum levels [29]. Further studies with larger sample sizes are required to clarify the regulation of 11-oxygenated androgens during pregnancy.

Plasma A4 and testosterone levels are significantly elevated during gestation. However, placental concentrations of A4 and testosterone were lower than those of 11-KA and 11-KT, respectively [17]. This indicates that the placenta makes only a minimal contribution to the elevation of classical androgens in the maternal blood. As mentioned above, androgen production in the maternal adrenal is unlikely to increase during gestation. Taken together, it is conceivable that the ovary is the main organ responsible for inducing the elevation of classical androgens in pregnant women. Consistent with this hypothesis, previous studies have reported that testosterone and A4 concentrations were not elevated in women with premature ovarian failure who became pregnant following in vitro fertilization and embryo transfer [30]. Ovarian androgen production might be induced by the repression of CYP19A1 expression in luteal cells during gestation [31]. Further studies are needed to identify the source of maternal androgens.

Based on studies of placental aromatase-deficient patients, it was hypothesized that the placental CYP19A1 protects the pregnant mother and fetus from androgen excess by eliminating placental androgens [8]. In addition, our study suggests that placental E2 could actively protect the mother and fetus by inhibiting the adrenal androgen production. This mechanism is likely necessary for preventing the excess production of 11-oxygenated androgens rather than classical androgens. Because 11-KT and 11-KA are weak substrates for CYP19A1 [13], it is conceivable that unlike classical androgens, they are not expended by aromatization in the placenta. In fact, aromatized 11-oxygenated androgens were undetectable in pregnant women [32]. However, 11-oxygenated androgens have recently been recognized as an important class of bioactive androgens in humans [23]. This class of androgens, including 11-KT, exhibits androgen receptor activity comparable to that of testosterone and is primarily produced in the adrenal gland. Recent studies suggest that they represent a major circulating androgen pool in women, and their levels are often elevated in girls and women with signs of virilization, such as premature adrenarche and polycystic ovary syndrome [33,34,35,36]. Nagasaki and colleagues also reported a case where 11-oxygenated androgens were overproduced by a maternal androgen-producing adrenal tumor, resulting in virilization of the mother and female fetus [37]. In contrast, A4 and T levels were constant in this patient. These facts indicate that adrenal production of 11-oxygenated androgens must be tightly regulated under conditions of elevated ACTH during gestation. Placental E2, produced via HSD17B1 activity, may therefore play an important role in maintaining maternal androgen homeostasis during gestation.

## 4. Materials and Methods

### 4.1. Human Placenta and Blood Sample Collection

Human placenta samples were collected in Asahikawa Medical University and Mori Hospital (Asahikawa, Hokkaido, Japan). Early (first-trimester) placenta (7–11 weeks gestation) were obtained from eight pregnant women (aged 28.2 ± 7.91 y) after elective interruption of a healthy pregnancy, whereas term placenta (37–40 weeks gestation) were collected from 8 pregnant women (aged 34.4 ± 3.16 y) immediately after delivery. All experiments were performed following the Declaration of Helsinki and were approved by the Asahikawa Medical University Research Ethics Committee (23178). Informed consent was obtained from all subjects.

Blood samples were collected in heparin-containing collection tubes from the median cubital vein of 2 healthy women volunteers as described [38], and 5 preterm mothers at the Asahikawa Medical University in 2024. Plasma was separated by centrifugation at 1000× *g* for 5 min. Other plasma samples were purchased from AllCells (Alameda, CA, USA) and ProMedDX (Norton, MA, USA). All plasma samples were collected under IRB-approved collection protocols and subjects’ informed consent. The donors were five non-pregnant women (aged 32.1 ± 7.80 y) and five pregnant women (aged 32.8 ± 3.90 y). Plasma samples were stored at −80 °C until assay.

### 4.2. Reverse Transcriptase-Polymerase Chain Reaction (RT-PCR) and Quantitative PCR (qPCR)

Total RNA from placental tissues was isolated using FastGene™ RNA Basic Kit (Nippon Genetics Co Ltd., Tokyo, Japan). RT-PCR and qPCR were performed as previously described [39]. cDNA was synthesized from the total RNA of each tissue using SuperScript III Reverse Transcriptase (Thermo Fisher Scientific; Waltham, MA, USA). cDNA from other human tissues (testis, ovary, and adrenal) were synthesized as described [13]. The reaction products of the RT-PCR assay were subjected to electrophoresis in a 1.25% agarose gel, and the resulting bands were visualized by staining with ethidium bromide (Figure S1). For qPCR, each gene was measured via real-time PCR using the LightCycler 480 (Roche Diagnostics, Mannheim, Germany). β-actin (human) was used as a reference gene. Each reaction was conducted in duplicate. As a negative control, template cDNA was replaced by PCR-grade water. Relative gene expression levels were determined by using the delta-delta Ct method. The primers used for PCR are listed in Table S1 and in previous studies [7,40,41].

### 4.3. Measurements of Steroid Hormones by Liquid Chromatography-Tandem Spectrometry (LC-MS/MS)

Steroid hormone levels in plasma samples were quantified by LC-MS/MS as previously described (ASKA Pharma Medical Co., Ltd., Kanagawa, Japan) [38,42]. As internal standards, E1, E2, testosterone, A4, 11-OHA, 11-OHT, 11KA, and 11-KT were added to a medium diluted with distilled water. The steroids were extracted with methyl *tert*-butyl ether (MTBE). After evaporating the MTBE layer to dryness, the extract was dissolved in 0.5 mL of methanol and then diluted with 1 mL of distilled water. The sample was applied to the OASIS MAX cartridge, which had been successively conditioned with 3 mL of methanol and 3 mL of distilled water. After the cartridge was washed with 1 mL of distilled water, 1 mL of methanol/distilled water/acetic acid (45:55:1, *v*/*v*/*v*), and 1 mL of 1% pyridine solution, the steroids were eluted with 1 mL of methanol/pyridine (100:1, *v*/*v*). After evaporation, the residue was reacted with 50 μL of mixed solution (80 mg of 2-methyl-6-delta-delta ct methodnitrobenzoic anhydride, 20 mg of 4-dimethylaminopyridine, 40 mg of picolinic acid, and 10 μL of triethylamine in 1 mL of acetonitrile) for 30 min at room temperature. After the reaction, the sample was dissolved in 0.5 mL of ethyl acetate/hexane/acetic acid (15:35:1, *v*/*v*), and the mixture was applied to a HyperSep Silica cartridge that had been successively conditioned with 3 mL of acetone and 3 mL of hexane. The cartridge was washed with 1 mL of hexane and 2 mL of ethyl acetate/hexane (3:7, *v*/*v*). E1, E2, testosterone, A4, 11-OHA, 11-OHT, 11-KA, and 11-KT were eluted with 2.5 mL of acetone/hexane (7:3, *v*/*v*). After evaporation, the residue was dissolved in 0.1 mL of acetonitrile/distilled water (2:3, *v*/*v*), and the solution was subject to LC-MS/MS.

### 4.4. Statistical Analyses

Statistical analyses were performed using the Wilcoxon rank-sum test with EZR ver. 1.70 (Easy R, Saitama Medical Center, Jichi Medical University, Saitama, Japan) to compare differences between two independent groups. Data are presented as box-and-whisker plots showing the median and interquartile range. A *p*-value < 0.05 was considered statistically significant.

## Figures and Tables

**Table 1:** Plasma steroid levels and ratios. Data are expressed as median (range). Lower limits of quantification (LLOQ) and pregnant/non-pregnant ratios of each steroid are also shown.

Steroids (ng/mL)	Non-Pregnant Women	Pregnant Women	LLOQ (ng/mL)	Pregnant/ Non-Pregnant
Androstenedione (A4)	1.0 (0.46–1.6)	3.0 (1.6–4.5)	0.01	2.82
Testosterone (T)	0.28 (0.13–0.34)	1.5 (0.68–2.2)	0.005	5.39
Estrone (E1)	0.02 (0.02–0.07)	3.9 (3.3–8.2)	0.005	154.9
Estradiol (E2)	0.02 (0.006–0.14)	22.9 (19.3–27.1)	0.005	545.2
11-KA	0.18 (0.09–0.25)	0.11 (0.07–0.30)	0.01	0.75
11-KT	0.4 (0.17–0.64)	0.22 (0.09–0.62)	0.005	0.65
11-OHA	1.1 (0.47–1.6)	0.50 (0.31–1.6)	0.01	0.64
11-OHT	0.08 (0.06–0.20)	0.04 (0.02–0.18)	0.005	0.62
				
**Ratios**				
T/A4	0.28 (0.18–0.35)	0.43 (0.38–0.56)		
E2/E1	1.0 (0.30–2)	5.7 (2.2–7.5)		
11-KT/11-KA	2.2 (1.1–4.1)	1.9 (1.3–2.1)		

## Data Availability

The original contributions presented in this study are included in the article/Supplementary Materials. Further inquiries can be directed to the corresponding author.

## Supplementary Materials

The following supporting information can be downloaded at: https://www.mdpi.com/article/10.3390/ijms27104290/s1.
