# Supplementary material for: The Expression of Placental 17β-Hydroxysteroid Dehydrogenase Genes Is Associated with the Elevation of Active Androgens and Estrogens in Pregnant Women, but Does Not Affect 11-Oxygenated C19 Steroids
Source: Int J Mol Sci. 2026 May 12;27(10):4290. doi: 10.3390/ijms27104290 (PMC13207966; doi:10.3390/ijms27104290)
Supplement: Supplementary file 1 [file ijms-27-04290-s001.zip › ijms-4238753-supplementary.pdf]

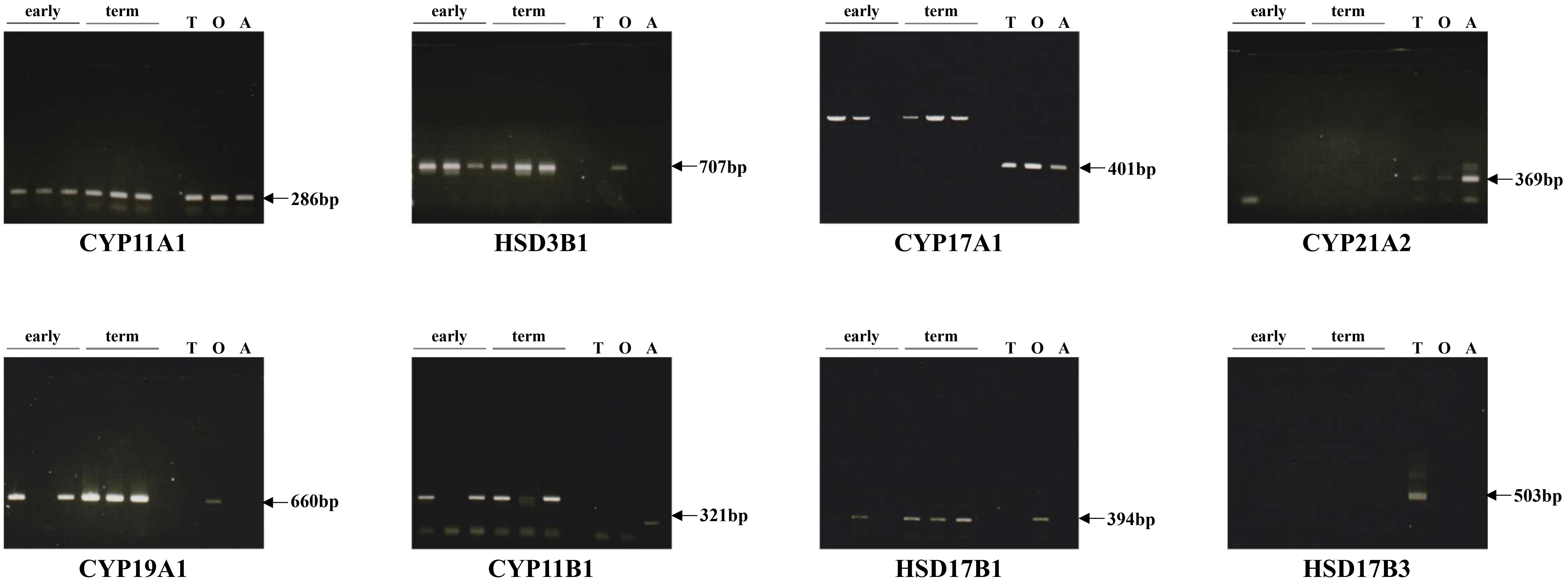

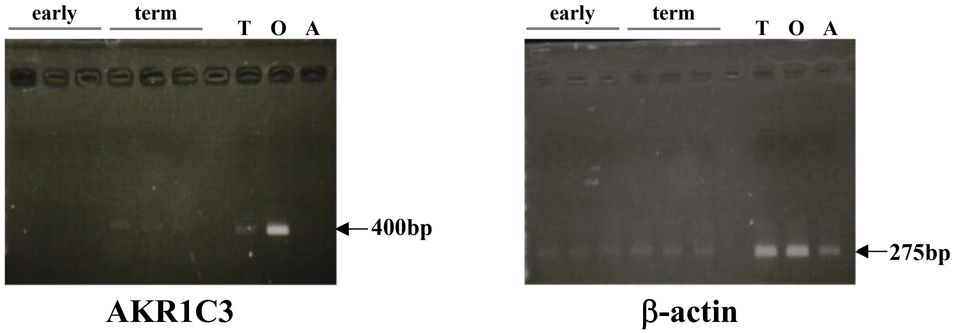

Figure S1. The whole images of RT-PCR analyses in Figure 2

Supplementary Table S1. Primers used in qPCR experiments

**primers**	**Forward primers**	**Reverse primers**
RT-PCR		
Human AKR1C3	F- atggcagtgtgaagagagaagac	R- tctagcaatttactccggttga
qPCR		
human CYP11A1	F- accagagacccataggagtcc	R- caacaggggcaaaaagttcttg
human HSD3B1	F- cggctaacgggtggaatctg	R- ccccatagatatacatgggtcgtaag
human HSD11B2	F- tgcttcaagacagagtcagtg	R- ggcatctacaactggggtga
human HSD17B1	F- ccttccaccgcttctaccaat	R- aagcgctcggtggtgaagta
human AKR1C3	F- agccaggtgaggaactttca	R- cctcccaggtggtacagaga
human HSD17B2	F- cacgaagccagtgcagataa	R- attgttgatcacagccccaca
human STARD3	F- gagcgatggtatcttgccg	R- ctgcaaaggattctgggggt
